# From Recession to Depression? Prevalence and Correlates of Depression, Anxiety, Traumatic Stress and Burnout in Healthcare Workers during the COVID-19 Pandemic in Greece: A Multi-Center, Cross-Sectional Study

**DOI:** 10.3390/ijerph18052390

**Published:** 2021-03-01

**Authors:** Sofia Pappa, Nikolaos Athanasiou, Nikolaos Sakkas, Stavros Patrinos, Elpitha Sakka, Zafeiria Barmparessou, Stamatoula Tsikrika, Andreas Adraktas, Athanasia Pataka, Ilias Migdalis, Sofia Gida, Paraskevi Katsaounou

**Affiliations:** 1 Department of Psychiatry, Imperial College London, London W12 0NN, UK; 2 Department of Respiratory Medicine, National and Kapodistrian University of Athens, 157 72 Athens, Greece; nikolaosathanasiou14@gmail.com (N.A.); nicksakkas1@gmail.com (N.S.); stavrosspatrinos@gmail.com (S.P.); elpithasakka@hotmail.com (E.S.); zafeiria.barmparessou@gmail.com (Z.B.); paraskevikatsaounou@gmail.com (P.K.); 3 School of Pharmacy and Biomolecular Science, University of Brighton, Brighton BN2 4AT, UK; 4 Respiratory Failure Clinic, Sotiria Hospital, 115 27 Athens, Greece; matatsik@yahoo.gr; 5 Pammakaristos Hospital, 111 44 Athens, Greece; andreas.adraktas@gmail.com; 6 Respiratory Failure Unit, Papanikolaou Hospital Thessaloniki, 570 10 Thessaloniki, Greece; patakath@yahoo.gr; 7 417 Army Equity Fund Hospital (NIMTS), 115 21 Athens, Greece; ilianmig@otenet.gr; 8 Trikala Hospital, 421 00 Trikala, Greece; sophia.gida@gmail.com; 9 Pulmonary and Respiratory Failure Department, First ICU, Evaggelismos Hospital, 106 76 Athens, Greece

**Keywords:** COVID-19, healthcare workers, Greece, mental health, depression, anxiety, traumatic stress, burnout

## Abstract

The COVID-19 pandemic has the potential to adversely affect the mental health of healthcare workers (HCWs). The public healthcare system in Greece was already facing serious challenges at the outset of the outbreak following years of austerity and an escalating refugee crisis. This multi-center, cross-sectional study aims to assess the levels and associated risk factors of anxiety, depression, traumatic stress and burnout of frontline staff in Greece. A total of 464 self-selected HCWs in six reference hospitals completed a questionnaire comprising sociodemographic and work-related information and validated psychometric scales. The proportion of HCWs with symptoms of moderate/severe depression, anxiety and traumatic stress were 30%, 25% and 33%, respectively. Burnout levels were particularly high with 65% of respondents scoring moderate/severe in emotional exhaustion, 92% severe in depersonalization and 51% low/moderate in personal accomplishment. Predictive factors of adverse psychological outcomes included fear, perceived stress, risk of infection, lack of protective equipment and low social support. The psychological burden associated with COVID-19 in healthcare professionals in Greece is considerable, with more than half experiencing at least mild mental health difficulties. Findings signal the need for immediate organizational and individually tailored interventions to enhance resilience and support wellbeing under pandemic conditions.

## 1. Introduction

In December 2019, a highly infectious acute respiratory syndrome caused by a novel coronavirus (SARS-CoV-2) emerged in Wuhan, China. By early March 2020, the World Health Organization (WHO) had declared COVID-19 a pandemic [1].

Previous experience from SARS and Ebola epidemics underscored the potential of such outbreaks to affect the mental health of the general population, as well as of patients and healthcare workers (HCWs) [2,3,4]. An early position paper in The Lancet [5], emphasized the central role of mental health scientific research in the international response to the COVID-19 pandemic and called for high-quality data on the mental health effects across the whole population and vulnerable groups such as healthcare professionals.

Inadequate personal equipment, physical exhaustion, nosocomial transmission and risk or infections of friends and relatives, stigma, isolation and loss of social support, the need to make ethically difficult decisions and adjust to drastic changes may all have dramatic effects on the physical and mental wellbeing of HCWs and compromise their resilience [6]. Previous reviews have explored the prevalence and factors associated with psychological outcomes in HCWs during past infectious disease outbreaks [7] and several studies have emerged during the current pandemic demonstrating the considerable occupational and psychological impact of this pandemic on the workforce across different countries and healthcare systems [8]. Subsequent rapid reviews further confirmed that, despite the heterogeneity of the included studies and some degree of variation in findings, HCWs are particularly vulnerable to mental health difficulties, including fear, anxiety, depression, insomnia, and burnout [9,10,11]. Furthermore, traumatic stress seems to be an important cause of psychological disability and post-traumatic stress disorder (PTSD) in the aftermath of a pandemic. In a recent review on healthcare workers facing outbreaks, fear of contagion, perceived stress, risk of infection, lack of protective equipment and low social support emerged as possible risk factors for psychological distress [12].

Greece has suffered epidemics before but was by and large spared following the most severe pandemic crisis in recent history, the 1918 Spanish flu. The SARS 2003 epidemic did not affect Greece while the HIV outbreak was confined to a specific subpopulation of injecting drug users [13]. Similarly, the impact of the West Nile virus and influenza A (H1N1) was limited [14]. Although their psychological impact on the Greek general population was not investigated, it was most likely low, though a study of healthcare workers revealed moderately high concern in over half of the sample during the H1N1 outbreak [15].

The first COVID-19 case in Greece was announced on 26th February 2020 and the first wave of the outbreak was mostly benign following the implementation of a successful lockdown during the initial phase of the crisis; restrictions were imposed early, and lockdown measures were largely adhered to by the public. However, studies conducted during this period in the general population showed high levels of depression and anxiety symptoms which were similar or higher compared to past assessments especially when compared to the period preceding the 2009 economic crisis, due to the already heightened prevalence rates amid the recession in the country [16,17]. A strong emotional impact of the epidemic was observed more often in women and in those with severe financial difficulties, and depressive symptoms were higher in the younger, in students and in those isolated due to symptoms or overexposed to media for COVID-19-related news [18]. Another study also confirmed the high prevalence of depressive symptoms in students during the same period [19].

Furthermore, the Greek public healthcare system was facing serious challenges at the dawn of the COVID-19 pandemic following more than a decade of economic recession and a difficult to contain refugee crisis; hence, levels of resilience and morale amongst HCWs were likely to be already compromised at the outset of this crisis [20]. However, to date, the full impact of the current unprecedented crisis on the psychological wellbeing of medical and nursing staff in Greece is yet to be established. Therefore, the aim of the current study was to examine the effects of the COVID-19 outbreak on the mental health of Greek frontline HCWs, which we hypothesized would be considerable, and particularly in relation to the prevalence and correlates of anxiety, depression, traumatic stress and burnout. Immediate interventions are essential in order to enhance psychological resilience and strengthen the healthcare systems’ capacity [21] and are of critical importance in view of the challenges faced during the considerably more fatal subsequent waves.

## 2. Materials and Methods

### 2.1. Study Design and Population

We conducted a cross-sectional study at six COVID-19 reference hospitals in Greece dedicated to treating hospitalized COVID-19 confirmed patients during the first wave of the pandemic from beginning of May 2020, to the end of June 2020. These hospitals were located in regions with higher transmission rates and mortality in Greece and frontline medical, nursing and allied healthcare professionals were asked to participate in this self-administered survey, following approval by the clinical research ethics committee of each site (Ethical Approval Number—198). Participants were self-selected and were provided with a link which enabled participation in the study after giving informed consent. The study was anonymous and confidential, and participants were allowed to terminate the survey at any time if they wanted. All HCWs who were working in frontline clinical services of these hospitals were eligible to participate with no other restrictions.

### 2.2. Questionnaire

Data collected in the self- reported survey questionnaire included socio-demographic information, medical history, lifestyle, work environment and psychometric scales assessing levels of fear, anxiety, depression, insomnia, traumatic stress and burnout:

- Socio-demographic and clinical factors: gender, age, occupation, medical and psychiatric history, smoking and recent vaccination history (influenza/*S. pneumoniae*).
- COVID-19 work-related factors: exposure to COVID-19 cases (no exposure, exposure to suspected or confirmed COVID-19 case), working on wards or departments dedicated exclusively to treating COVID-19 patients, access to adequate information and adequate personal protective equipment for prevention COVID-19 infection, if participants were considered “high risk” depending on age and comorbidities and if they tested positive, had symptoms of COVID-19 and/or had to self-isolate.
- COVID-19 emergency-related worries: “I am worried of getting infected by COVID-19”, “I am worried of transmitting COVID-19 to family and friends/others”, “I am worried COVID-19 will have an impact on my mental health/my job/my ability to care for individuals/my family and friends/my financial status/on society”). Items were rated on a 5-point Likert-type scale, ranging from 0 (not at all) to 4 (extremely).
- COVID-19 emergency-related psychological factors, including questions concerning sleep difficulties, experiencing nightmares or flashbacks and self-harming behavior or suicidal ideation. Responses were rated on a 4-point scale ranging from 0 (No, not at all) to 1 (Yes, less than before), 2 (Yes, same as before) and 3 (Yes, more than before). Moreover, the participants were asked if they would seek professional wellbeing advice and support if needed and if they are aware how to access it.

### 2.3. Psychometric Scales

- Patient Health Questionnaire-9 (PHQ-9) is a 9-item self-administered screening tool for depression [22]. The scale investigates symptom severity over the past two weeks. Items are rated on a 4-point Likert type scale, ranging from 0 (not at all) to 3 (nearly every day). Total scores range between 0 and 27; scores of 0–4 are regarded as “minimal or none”, 5–9 as “mild”, 10–14 as “moderate”, 15–19 as “moderately severe”, and 20–27 as “severe”. The recognized cut-off point of 10 or greater corresponds to moderate to severe symptomatology indicative of a clinically significant problem. The scale has been validated in Greek [23].
- General Anxiety Disorder-7 (GAD-7) is a 7-item self-reported anxiety scale evaluating symptom severity in the preceding two weeks [24]. Items are rated on a 4-point Likert-type scale, ranging from 0 (not at all) to 3 (nearly every day). Total scores range between 0 and 21. Total scores of 0–4 were regarded as “not at all”, 5–9 as “mildly”, 10–14 as “moderately” and 15 as “severely”. The scale has been validated in Greek [25].
- Impact of Event Scale-Revised (IES-R) is a validated 22-item self-report that measures the subjective psychological distress in response to traumatic events [26,27]. Respondents are asked to indicate on a 5-point Likert scale ranging from never (score 0) to often (score 4) how frequently each symptom was experienced during the past week. It has 3 subscales (Intrusion, Avoidance and Hyperarousal), which are closely associated with post-traumatic stress disorder (PTSD) symptom. Total scores range between 0 and 88 and are graded for severity from normal (0–23), mild (24–32), moderate (33–36) to severe psychological distress (>37). A cut-off score of 24 is commonly used to define PTSD of a clinical concern [28]. The Greek version used has shown good psychometric features [29].
- Burnout: Maslach Burnout Inventory (MBI) is a 22-item questionnaire which assesses three dimensions: emotional exhaustion (EE, 9 items), depersonalization (DE, 5 items) and personal accomplishment (PA, 8 items) [30]. Higher scores in the emotional exhaustion and depersonalization dimensions indicate more severe burnout, whereas higher scores in the personal accomplishment subscale indicate less burnout. Cut-offs for moderate and severe emotional exhaustion were ≥17 and ≥27, for moderate and severe depersonalization ≥7 and ≥13, and for moderate and severe reduced personal accomplishment ≤38 and ≤21. The Greek translation of the scale was employed [31].
- A numerical fear rating scale (NFRS) was used to measure the level of fear in the study which has been reported to have good reliability and validity [32]. It is a segmented numeric version of the visual analog scale (VAS) in which a respondent selects a whole number (0–10 integers) that best reflects the intensity of their fear. Higher scores indicate greater fear as follows: 0 for no fear, 1–3 for mild fear, 4–6 for moderate fear, 7–9 for severe fear, 10 for extreme fear.

### 2.4. Statistical Analysis

Statistical analysis was performed using Stata v12.0 (StataCorp. 2011. Stata Statistical Software: Release 12. College Station, TX: StataCorp LP, Athens, Greece). Descriptive statistics were used to present sociodemographic and other COVID-related information and continuous outcome variables including, fear, anxiety, depression, traumatic stress and burnout; categorical variables were expressed as absolute values (percentages) and continuous variables as mean values ± (standard deviation). Student’s *t*-test and Analysis of Variance (ANOVA) were used to examine the association between continuous variables and Pearson’s chi-square test or Fisher’s exact was used to evaluate categorical variables. Multivariable logistic regression was used to determine independent associations of binary outcomes. Two-tailed *p* values of less than 0.05 were deemed statistically significant.

## 3. Results

### 3.1. Participant Characteristics

A total of 464 healthcare workers participated in the study with a mean age of 41.37 (SD:11). The sample was predominantly female (68%), nurses (43%), married (49%), with higher education (77%) and directly involved in the care of COVID-19 patients (87%). Table 1 summarises the sociodemographic and basic clinical information of the sample.

**Figure 1:**
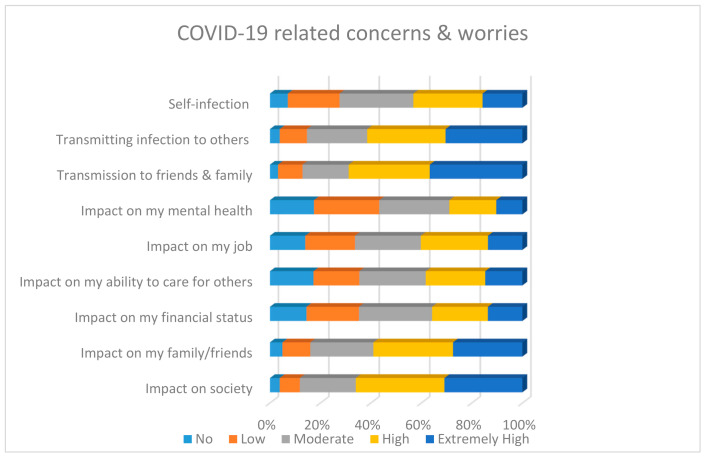
COVID-19 related self-reported concerns about risk of infection and impact of pandemic.

**Figure 2:**
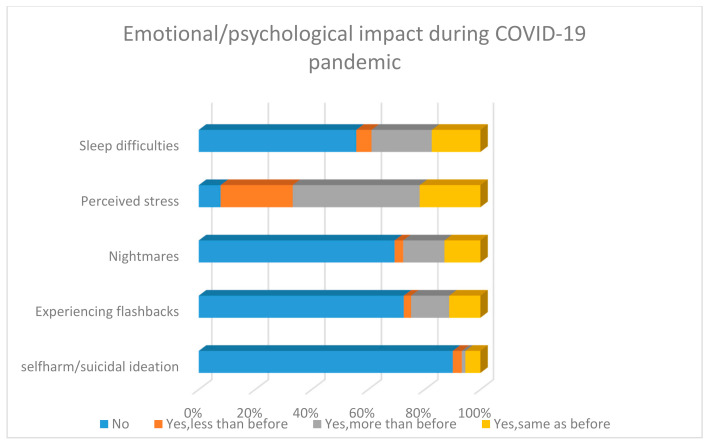
Self-reported emotional impact during COVID-19 pandemic.

Most participants were worried about infecting others, particularly friends and family and the impact of the pandemic on friends, family and society as shown in Figure 1. Furthermore, a fair proportion of respondents reported experiencing far more self-reported sleeping difficulties, nightmares, perceived stress and flashbacks compared to before the start of the pandemic (Figure 2). In addition, a very small minority reported the presence/increase of suicidality.

Only a combined 30% (yes/probably yes) indicated that they would like more information/access to psychological support, although 68% indicated that they knew where to find help for their mental health if needed.

### 3.2. Psychometric Scales Outcomes

**Figure 3:**
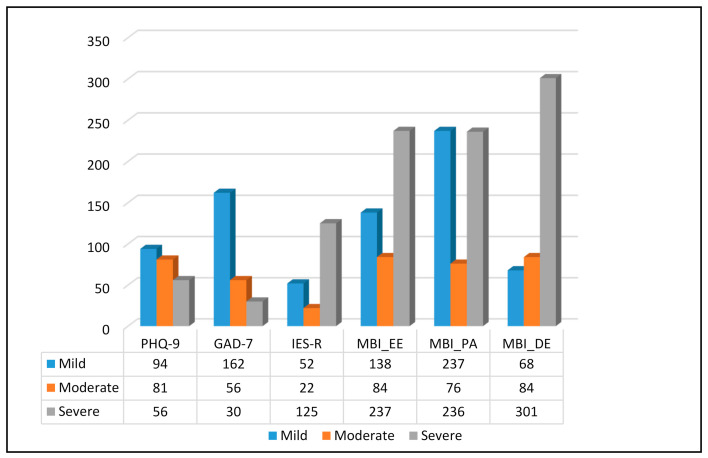
Number of participants with mild, moderate and severe symptoms of depression, anxiety, stress and burnout. PHQ-9 = Patient Health Questionnaire-9; GAD-7 = General Anxiety Disorder-7; IES-R = Impact of Event Scale-Revised; MBI_EE = Maslach Burnout Inventory, Emotional Exhaustion; MBI_PA = Maslach Burnout Inventory, Personal Accomplishment; MBI_DE = Maslach Burnout Inventory, Depersonalization.

A significant proportion of HCWs reported at least mild symptoms of depression, anxiety, traumatic stress and/or burnout. The total levels of severity as well as by sex and occupation are illustrated in Table 2 and Table 3 and Figure 3. Additional normality and regression analysis tests are available under Supplementary data.

The proportion of healthcare workers with symptoms of moderate/severe depression were 30.18% and moderate/severe anxiety were 25.66%. The logistic regression analysis showed that higher level of perceived stress due to COVID-19 (OR: 15.6, *p* = 0.018), fear (OR: 1.22, *p* = 0.006), lack of protective equipment (67–77%—*p* < 0.01), lack of social support (OR: 0.29, *p* = 0.002) and more frequent nightmares (OR: 2.6, *p* = 0.02) and flashbacks (OR: 2.8, *p* = 0.008) were significantly associated with a higher likelihood of exhibiting symptoms of depression. A higher level of perceived stress (OR: 2.95, *p* = 0.025) and fear (OR: 1.3, *p* < 0.001), flashbacks (OR: 3, *p* = 0.001), the presence of COVID-19 symptoms (OR: 2, *p* = 0.018) and higher education level (OR: 0.56, *p* = 0.046) were significant predictors of anxiety.

A considerable proportion experienced traumatic stress with 45% reporting symptoms above the cut-off for possible post-traumatic stress disorder and 33% reporting moderate and severe stress. Traumatic flashbacks (OR: 4–4.8, *p* < 0.001), financial worry (OR: 0.375, *p* = 0.044), low social support (OR: 5, *p* = 0.012) and experiencing more nightmares (OR: 3.7, *p* = 0.001) were significant predictors of traumatic stress.

Furthermore, HCWs reported high levels of burnout in all three dimensions: emotional exhaustion was moderate in 21.35% and high in 44.01% and depersonalization was high in 92.22%, while personal accomplishment was low in 26.55% and moderate and high in 24.12% and 49.34% respectively. The regression analysis model revealed that perceived stress (b = 6.6–9, *p* < 0.01), traumatic flashbacks (b = 6.2–8.2, *p* < 0.001), suicidality (b = −6.1, *p* = 0.021) and severe worry about the impact of the pandemic on society (b = 6.2, *p* = 0.036) were significant predictors of emotional exhaustion. Worry of self-infection (b = 3.2, *p* = 0.034), high infection risk group (b = 2.5, *p* = 0.017) and lack of protective equipment (b = −3.2, *p* = 0.003), alongside perceived stress (b = 4.8–5.8, *p* < 0.001) and traumatic flashbacks (b = 2.5–4, *p* < 0.03), were significantly associated with depersonalization. Worry of friend/family infection (b = 8.1–10.4, *p* < 0.02), female gender (b = 4.3, *p* < 0.001), perceived stress (b= (−5.5)–(−10.3), *p* < 0.05), traumatic flashbacks (b = −4.1, *p* = 0.02) and awareness about support seeking (b = 2.5, *p* = 0.037) were found to correlate with a lower sense of personal accomplishment.

## 4. Discussion

To our knowledge, this is the first multi-center study to report on the prevalence and correlates of depression, anxiety and burnout in the medical workforce in Greece during the COVID-19 pandemic. The findings revealed high levels of mental health symptoms among healthcare workers during the early phase of the outbreak despite its relatively benign course at the time.

As mentioned, the public healthcare system was already fragile and compromised by a decade of austerity and cuts and an increasingly unmanageable refugee crisis. Hence, prevalence rates in our sample are generally at the higher end of psychological outcomes previously reported among HCWs across different countries and regions, though some of these may have experienced considerably higher transmission rates and pressures on healthcare services at the time. Furthermore, our analysis did not overall demonstrate important gender, age, occupational or regional differences as had been the case in previous studies, but underpinned a number of potential predictive or mediating factors. Again, it is important to note that comparisons between studies have to made with caution given the inherent heterogeneity across studies as different assessment scales were utilized for population screening and different cut-offs were applied [33].

Regardless, however, of the criteria applied for case definition, our study adds to the existing evidence regarding the need for early detection and effective treatment not only of the more severe but also the milder clinical mental health symptoms or sub-threshold syndromes in HCWs before they evolve to more complex and enduring psychological reactions.

### 4.1. Depression and Anxiety

Over 50% of participants reported at least mild depressive symptoms; of these, 30% were moderate to severe. The proportion of healthcare workers with symptoms of at least mild anxiety were 61.5%, with 25% reporting moderate to severe symptoms. Higher levels of fear and perceived stress, more frequent nightmares and flashbacks and lack of protective equipment and social support were significantly associated with a higher likelihood of exhibiting symptoms of depression. Likewise, higher levels of fear and perceived stress, more frequent flashbacks, the presence of COVID-19 symptoms and a higher education level (OR: 0.56, *p* = 0.046) were significant predictors of anxiety.

Overall, anxiety symptoms were overall higher compared to depression; a finding consistent across most studies to date. Our own rapid review with meta-analysis on 12 studies performed in China and 1 study performed in Singapore showed similar prevalence rates of depression (22.8%), anxiety (23.2%) and insomnia (38.9%) in HCWs [10]. Pooled prevalence of depression and anxiety were 28% and 33%, respectively, in a subsequent meta-analysis; rates were highest among patients with pre-existing conditions and COVID-19 infection (56% and 55%) and were overall similar between healthcare workers and the general public. However, studies from a number of countries such as China, Italy, Turkey, Spain and Iran reported higher than pooled prevalence among healthcare workers and the general population [9]. Common risk factors included being female and a nurse, having lower socioeconomic status, high infection risk and social isolation, and protective factors included having sufficient medical resources, protection and up-to-date accurate information. Depressive symptoms ranged between 27.5–50.7%, severe anxiety symptoms were reported in 45% and insomnia symptoms in 34–36% of HCW in the systematic review by Preti et al. [34].

In our study, the levels of depression and anxiety in HCWs were higher or similar to those reported in the general Greek population around the same period of time, although these may be difficult to compare due to the different methodologies used across studies. Clinical depression was present in 9.3% and increased anxiety in more than 45% of the sample in a study by Fountoulakis et al. [17], whereas suicidal thoughts increased in 10.4% and decreased in 4.4%. In another study, a significant proportion reported moderate to severe depressive symptoms (22.8%), moderate to severe anxiety symptoms (77.4%) or COVID-19-related fear (35.7%), with women scoring altogether significantly higher than men [16].

A further study conducted in early April showed that a strong emotional impact of the epidemic was more often observed in women and in those with severe financial difficulties [18]. Depressive symptoms were higher in the younger, in students, in those with a stronger emotional impact, in those isolated due to symptoms, and those overexposed to media for COVID-19-related news. Students were also likely to report depression independently of age: major depression was present in 12.43% with 13.46% experiencing severe distress [35]. Risk factors were female sex and a history of self-injury and suicidal attempts.

Interestingly, some of the findings in the general population in Greece resemble those in Italy during the same period of time despite the significantly higher transmission rate in the latter at the time. A population study in 2291 participants demonstrated that 32% of the participants reported high levels of anxiety, 41.8% psychological distress, 7.6% PTSD-type symptoms and 57% poor sleep quality [36,37]. Young age, female gender and increased fear of infection were risk factors for sleep and mood problems. Findings are generally not very dissimilar across different populations in Europe while female sex and younger age are often identified as significant predictors [38,39].

### 4.2. Traumatic Stress

A considerable proportion of HCWs experienced traumatic stress with one third of the sample reporting moderate to severe stress and a total of 45% reporting symptoms above the cut-off for possible post-traumatic stress disorder. Furthermore, low social support and financial worry were significant predictors of traumatic stress.

The prevalence of traumatic stress observed in this study is at the higher end of rates reported previously. In an earlier online survey involving 270 participants, Greek healthcare professionals appeared to be moderately stressed from the COVID-19 crisis, with women scoring significantly higher than men on all clinical scales; this was not the case in our sample [40]. Furthermore, criteria for a probable post-traumatic stress disorder diagnosis were met by 16.7% (21.7% of women; 5.1% of men).

A systematic review which included 44 studies showed that between 11–73.4% of HCWs experienced PTSD-type symptoms during the latest outbreaks of SARS, MERS, Ebola and Influenza A, with symptoms lasting for at least 1–3 years in 10–40%. [34]. The vast variation among these results could be explained by differences in contagion rates, pressure and preparedness of healthcare systems, incidence of mediating factors and access to occupational and psychological support. In addition, risk factors, such as female gender, younger age, occupation, lack of adequate protective equipment and exposure to infected people, have been found to be associated with higher levels of traumatic stress and PTSD in previous epidemics [41,42].

Regarding the COVID-19 outbreak, available studies show a significant impact of COVID-19 trauma and stress-related symptoms in the general population and in patients [43,44,45]. The reported prevalence of clinically relevant traumatic stress in HCWs ranged from 7.4 to 35% [44,45,46,47,48]. Female age, younger age, occupation, exposure to infected people, poor social support, insomnia and physical symptoms are some common risk factors for traumatic symptoms in HCWs [47,49].

A recent meta-analysis showed that PTSD features among HCWs were more frequent in MERS (40.7%) than in SARS (16.7%) and COVID-19 (7.7%), which could relate to the higher mortality rates of MERS [50]. Similarly, the frequency of PTSD features in HCW exposed to SARS/MERS/COVID-19 appeared lower (20.7%) than in the general population with SARS/MERS infection (32.5%) [50,51]. Having said that, PTSD symptoms usually have a delayed onset following the traumatic experience, and it may be too early to evaluate the full effects in the case of COVID-19 pandemic as has been the experience from previous epidemics [52]. Future studies are needed to evaluate the long-term trajectories of trauma and stress-related symptoms in HCWs exposed to COVID-19.

### 4.3. Burnout

HCWs recorded particularly high levels of burnout with 65% reporting moderate to high emotional exhaustion and 92% scoring high on depersonalization, while personal accomplishment was low in 26%, moderate in 24% and high in 49%. According to the regression model, increased levels of perceived stress and flashbacks were significant predictors of all three dimensions. Furthermore, suicidality and concern about the impact of the pandemic on society were significantly associated with emotional exhaustion and being in the high-risk group, worry of self-infection and lack of protective equipment were associated with higher levels of depersonalization. Interestingly, female sex, physician status and worrying about infection of friends/family correlated with lower rates of personal accomplishment.

In fact, burnout is already high among physicians in ordinary times (with prevalence rates up to or over 50%) [53] and was a frequently associated feature during previous epidemics particularly for HCWs working long hours [54]. During the current pandemic, prevalence of burnout among health professionals has overall attracted less attention compared to other psychological outcomes, but a number of studies have confirmed the presence of considerable emotional exhaustion and sense of reduced accomplishment [55,56]. Again, the noted variation in reported figures may be explained by socioeconomic and cultural differences alongside differences in preparedness and infrastructure of healthcare systems.

In a study by Giusti et al. [57] that evaluated the psychological impact of COVID-19 pandemic on HCWs in Italy—one of the harder hit regions during the initial stages of the outbreak—moderate to severe levels of emotional exhaustion were present in 67% and depersonalisation in 26% of the sample, while reduced personal accomplishment was recorded in more than 60% of the sample. In this study, predictors of all three components of burnout were long work hours, psychological comorbidities, fear of infection and perceived support by friends. Predictors of both emotional exhaustion and depersonalization were female gender, being a nurse, working in the hospital and being in contact with COVID-19 patients. Interestingly, in our study female gender was only associated with a reduced sense of personal accomplishment as women are more likely to experience higher levels of work–family conflict. Furthermore, the bidirectional association between anxiety/depressive symptoms and burnout syndrome in healthcare workers is well established. Thus, burnout prevalence is often associated with the presence of mood symptoms, while undetected and untreated distress and burnout can lead to long-term psychiatric complications in this population, as reported in a recent paper on healthcare workers facing the COVID-19 pandemic in Italy [58].

Overall, the above results have important implications for both staff wellbeing and the capacity and efficiency of healthcare systems. Burnout is associated with physical and psychological long-term negative consequences for physicians and other healthcare professionals, resulting in increased sick leave, absenteeism, reductions in work hours, medical errors, road accidents, various mental health concerns and suicidality [59]. Self-reported suicidal ideation and behavior was low in our sample, but physicians are already at an increased risk of suicide compared to the general population and there have been already reports of suicides of healthcare professionals faced with accumulated psychological pressure and intense fear of dying during this outbreak [60].

Hence, both organizational solutions and individual-focused interventions are required to support wellbeing and prevent the development of burnout and other mental health problems [61]. Provision of adequate protective equipment and priority vaccination alongside appropriate specialized training and clear communication may increase confidence and minimize fear of infection to self and others which appeared high in our sample. Managing workload and exhaustion by allowing for sufficient rest and sleep (limitation of shift hours, access to rest areas, enhancing frontline resources and workforce) is essential. Much can be also done in providing timely and appropriately tailored mental health support such as online psycho-education, chat lines, small group peer support, mindfulness and remotely delivered psychological therapies where needed. As noted previously, participants in our study also indicated that they are unlikely to proactively seek help or access services; thus, provision of these interventions in-house as part of an assertive organizational approach could decrease stigma and improve access.

### 4.4. Limitations

The study has some key limitations. It was a cross- sectional online survey involving an auto-selected sample thus not allowing for causal inferences to be made which limited our understanding of potential risk factors. The assessment of mental health symptoms was performed using self-reported instruments and may vary from clinical or specialist interviews as reported difficulties may not necessarily translate to a clinical syndrome. The total number of participants and the inclusion of different occupational groups from multiple sites, albeit more representative, introduces greater heterogeneity of the sample and limits the generalizability of the results. Moreover, longitudinal studies are needed to examine the trajectories of the mental health outcomes including PTSD which can often have a delayed onset. Finally, the lack of information about the baseline mental health status and previous history in the sample represents an important limitation given that subjects with previous mental health problems exposed to COVID-19 pandemic-related stress and/or infection may experience a higher mental health burden [62].

## 5. Conclusions

Overall, the study results confirmed the potential of this pandemic to adversely affect the psychological wellbeing of healthcare workers, demonstrating high prevalence rates of depression, anxiety, traumatic stress and burnout among Greek frontline staff. Findings can help to quantify staff support needs and inform tailored interventions under pandemic conditions that enhance resilience and mitigate vulnerability, particularly in light of the high levels of burnout and low morale observed.

## Figures and Tables

**Table 1:** Sample characteristics.

Age	*N*	Mean ± SD
Male/Female	464	41.37 ± 11
**Sex**	***N***	**%**
Male	145	31.25
Female	319	68.75
**Marital Status**		
Married	228	49.14
Single/Divorced/Widowed	236	50.86
**Educational Level**		
Secondary education	107	23.06
Higher education	357	76.94
**Occupation**		
Doctor	179	38.58
Nurse	200	43.10
Other	85	18.32
**Work Department**		
COVID Department	89	19.18
Pulmonary Clinic	83	17.89
Internal Medicine Department	56	12.07
ICU	73	15.73
Emergency Department	74	15.95
Other	89	19.18
**Direct Care of COVID-19 Patients**		
No	57	12.31
Yes	407	87.69
**COVID-19 Status**		
COVID-19 disease	5	1.08
Quarantine	47	10.13
Neither	412	88.79
**Experience of COVID-19 symptoms**		
No	364	78.45
Yes	100	21.55
**High-risk group for COVID-19**		
No/Maybe no	311	67.03
I am not sure	58	12.50
Yes/Maybe yes	95	20.47
**Compliance with recommended measures**		
No	118	25.43
Yes	346	74.57
**Sufficient personal protective equipment**		
No/Maybe no	114	24.57
I am not sure	64	13.79
Yes/Maybe yes	286	61.64
**Sufficient information from hospital authorities**		
No/Maybe no	112	24.14
I am not sure	84	18.10
Yes/Maybe yes	268	57.75
**Smoking status**		
Current smoker	155	33.41
Never smoker	220	47.41
Ex-smoker	89	19.18
**Influenza Vaccination**		
No	235	50.65
Yes	229	49.35
**Pneumococcal Vaccination**		
No	395	85.13
Yes	69	1487

**Table 2:** Psychometric scale outcomes: means and level of severity by sex and total. PHQ-9 = Patient Health Questionnaire-9; GAD-7 = General Anxiety Disorder-7; IES-R = Impact of Event Scale-Revised; MBI_EE = Maslach Burnout Inventory, Emotional Exhaustion; MBI_PA = Maslach Burnout Inventory, Personal Accomplishment; MBI_DE = Maslach Burnout Inventory, Depersonalization.

	***N* (%)**			
**PHQ-9**	**Male**	**Female**	**Total**	***p*-Value**
No/Minimum	71 (50.35)	152 (48.56)	223 (49.12)	0.835
Mild	25 (17.73)	69 (22.04)	94 (20.70)	
Moderate	27 (19.15)	54 (17.25)	81 (17.84)	
Higher moderate	14 (9.93)	27 (8.63)	41 (9.03)	
Severe	4 (2.84)	11 (3.51)	15 (3.30)	
**GAD-7**				
No stress	60 (43.17)	114 (36.42)	174 (38.50)	0.468
Mild	47 (33.81)	115 (36.74)	162 (35.84)	
Moderate	22 (15.83)	64 (20.45)	56 (19.03)	
Severe	10 (7.19)	20 (6.39)	30 (6.64)	
**IES-R**				
No stress	83 (59.29)	152 (51.70)	235 (54.15)	0.374
Mild	13 (9.29)	39 (13.27)	52 (11.98)	
Moderate	8 (5.71)	14 (4.76)	22 (5.07)	
Severe stress	36 (25.71)	89 (30.27)	125 (28.80)	
**MBI_EE**				
Low	48 (33.33)	90 (28.57)	138 (30.07)	0.430
Moderate	28 (19.44)	56 (17.78)	84 (18.30)	
High	68 (47.22)	169 (53.65)	237 (51.63)	
**MBI_PA**				
Low	65 (46.76)	172 (55.48)	237 (52.78)	0.007
Moderate	18 (12.95)	58 (18.71)	76 (16.93)	
High	56 (40.29)	80 (25.81)	136 (30.29)	
**MBI_DE**				
Low	26 (18.31)	42 (13.50)	68 (15.01)	0.153
Moderate	20 (14.08)	64 (20.58)	84 (18.54)	
High	96 (67.61)	205 (65.92)	301 (66.45)	
	**Mean ± Std. Error**			
	**Male**	**Female**	**Total**	***p*-Value**
**PHQ-9**	6.41 ± 0.50	6.72 ± 0.34	6.63 ± 0.28	0.6110
**GAD-7**	6.05 ± 0.40	6.78 ± 0.27	6.55 ± 0.22	0.1309
**IES-R**	22.42 ± 1.71	26.26 ± 1.23	25.02 ± 1.00	0.0734
**MBI_EE**	26.22 ±1.04	28.93 ± 0.76	28.08 ± 0.62	0.0421
**MBI_PA**	35.62 ± 1.10	39.89 ± 0.64	38.57 ± 0.56	0.0004
**MBI_DE**	14.05 ± 0.60	14.01 ± 0.41	14.02 ± 0.34	0.4802

**Table 3:** Psychometric scale outcomes: means and level of severity for physician and nurses. PHQ-9 = Patient Health Questionnaire-9; GAD-7 = General Anxiety Disorder-7; IES-R = Impact of Event Scale-Revised; MBI_EE = Maslach Burnout Inventory, Emotional Exhaustion; MBI_PA = Maslach Burnout Inventory, Personal Accomplishment; MBI_DE = Maslach Burnout Inventory, Depersonalization.

	***N* (%)**		
**PHQ-9**	**Male**	**Female**	***p*-Value**
No/Minimum	88 (50.57)	103 (52.55)	0.030
Mild	31 (17.82)	50 (25.51)	
Moderate	35 (20.11)	26 (13.27)	
Higher moderate	18 (10.34)	10 (5.10)	
Severe	2 (1.15)	7 (3.57)	
**GAD-7**			
No stress	67 (39.41)	82 (41.21)	0.726
Mild	65 (38.24)	76 (38.19)	
Moderate	30 (17.65)	28 (14.07)	
Severe	8 (4.71)	13 (6.53)	
**IES-R**			
No stress	99 (58.58)	108 (58.38)	0.545
Mild	15 (8.88)	24 (12.97)	
Moderate	8 (4.73)	10 (5.41)	
Severe stress	47 (27.81)	43 (23.24)	
**MBI_EE**			
Low	54 (30.34)	64 (32.32)	0.917
Moderate	37 (20.79)	40 (20.20)	
High	87 (48.88)	94 (47.47)	
**MBI_PA**			
Low	87 (50.00)	121 (62.37)	0.025
Moderate	25 (14.37)	28 (14.43)	
High	62 (35.63)	45 (23.20)	
**MBI_DE**			
Low	24 (13.71)	30 (15.23)	0.374
Moderate	31 (17.71)	45 (22.84)	
High	120 (68.57)	122 (61.93)	
	**Mean ± Std. Error**		
	**Doctors**	**Nurses**	***p*-Value**
**PHQ-9**	6.44 ± 0.44	5.85 ± 0.42	0.3295
**GAD-7**	6.31 ± 0.34	6.22 ± 0.33	0.8500
**IES-R**	23.24 ± 1.57	23.28 ± 1.50	0.9857
**MBI_EE**	27.67 ± 1.00	27.31 ± 0.93	0.7947
**MBI_PA**	36.84 ± 0.89	40.87 ± 0.87	0.0013
**MBI_DE**	14.25 ± 0.52	13.01 ± 0.51	0.0882

## Data Availability

The data presented in this study are available on request from the corresponding author.

## Supplementary Materials

The following are available online at https://www.mdpi.com/1660-4601/18/5/2390/s1, Figure S1: PHQ9 logistic regression model, Figure S2: GAD7 logistic regression model, Figure S3: MBI_EE linear regression, Figure S4: MBI_PA linear regression, Figure S5: MBI_DE linear regression, Figure S6: IESR logistic regression, Table S1: Skewness/Kurtosis tests for Normality.
