# Supplementary material for: From Recession to Depression? Prevalence and Correlates of Depression, Anxiety, Traumatic Stress and Burnout in Healthcare Workers during the COVID-19 Pandemic in Greece: A Multi-Center, Cross-Sectional Study
Source: Int J Environ Res Public Health. 2021 Mar 1;18(5):2390. doi: 10.3390/ijerph18052390 (PMC7967750; doi:10.3390/ijerph18052390)

Table S1. Skewness/Kurtosis tests for Normality

Variable	Obs	Pr(Skewness)	Pr(Kurtosis)	------- joint ------ adj chi2(2)	Prob>chi2
age	464	0.0762	0.0000	66.10	0.0000
covid_fear	463	0.8370	0.0000	49.56	0.0000
PHQ9	454	0.0000	0.9910	34.87	0.0000
GAD7	452	0.0000	0.4474	18.39	0.0001
MBI_EE	459	0.0012	0.0000	22.96	0.0000
MBI_PA	449	0.0000	0.1745	25.59	0.0000
MBI_DE	453	0.0000	0.0000	29.94	0.0000
IESR_total	434	0.0000	0.0026	27.38	0.0000

Figure S1. PHQ9 logistic regression model

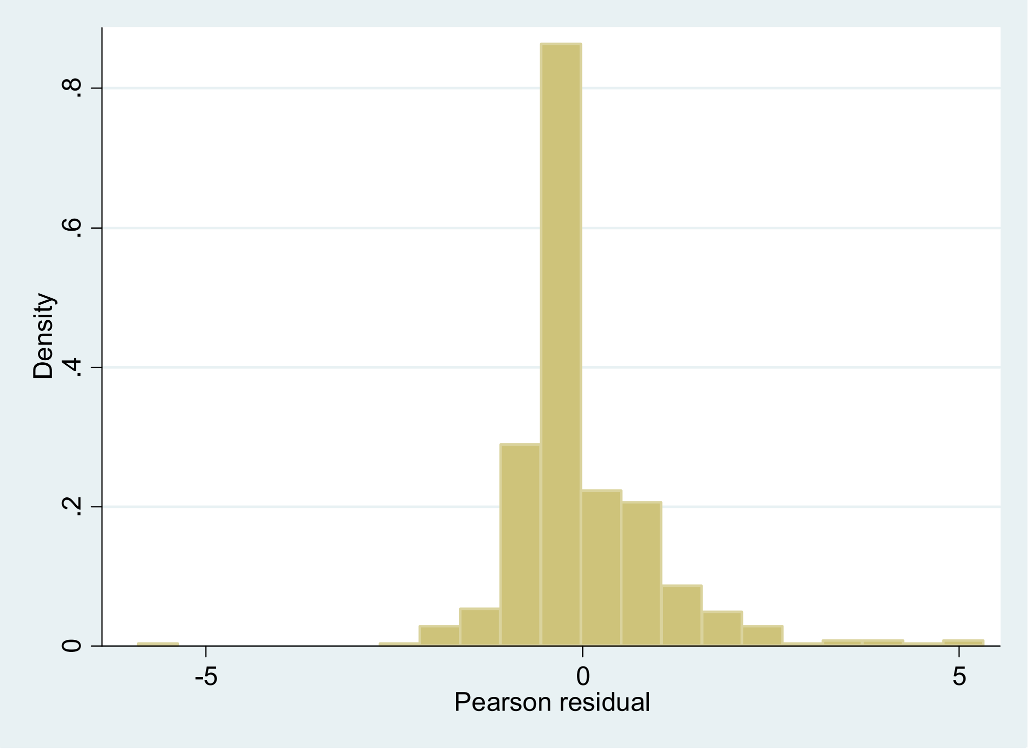

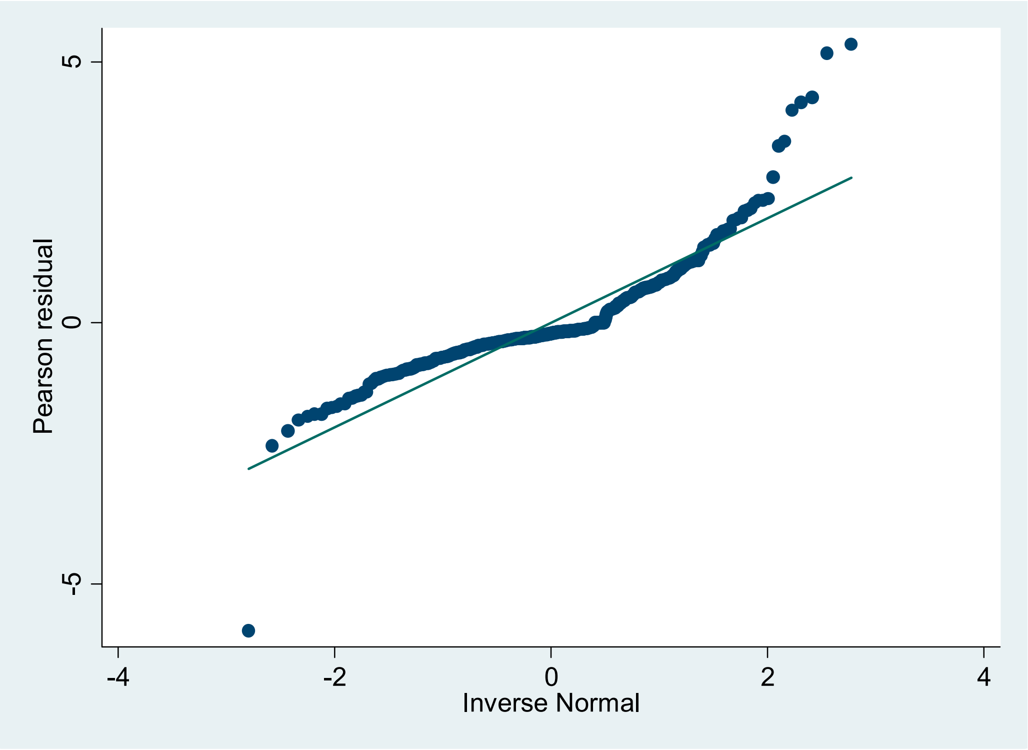

Figure S2. GAD7 logistic regression model

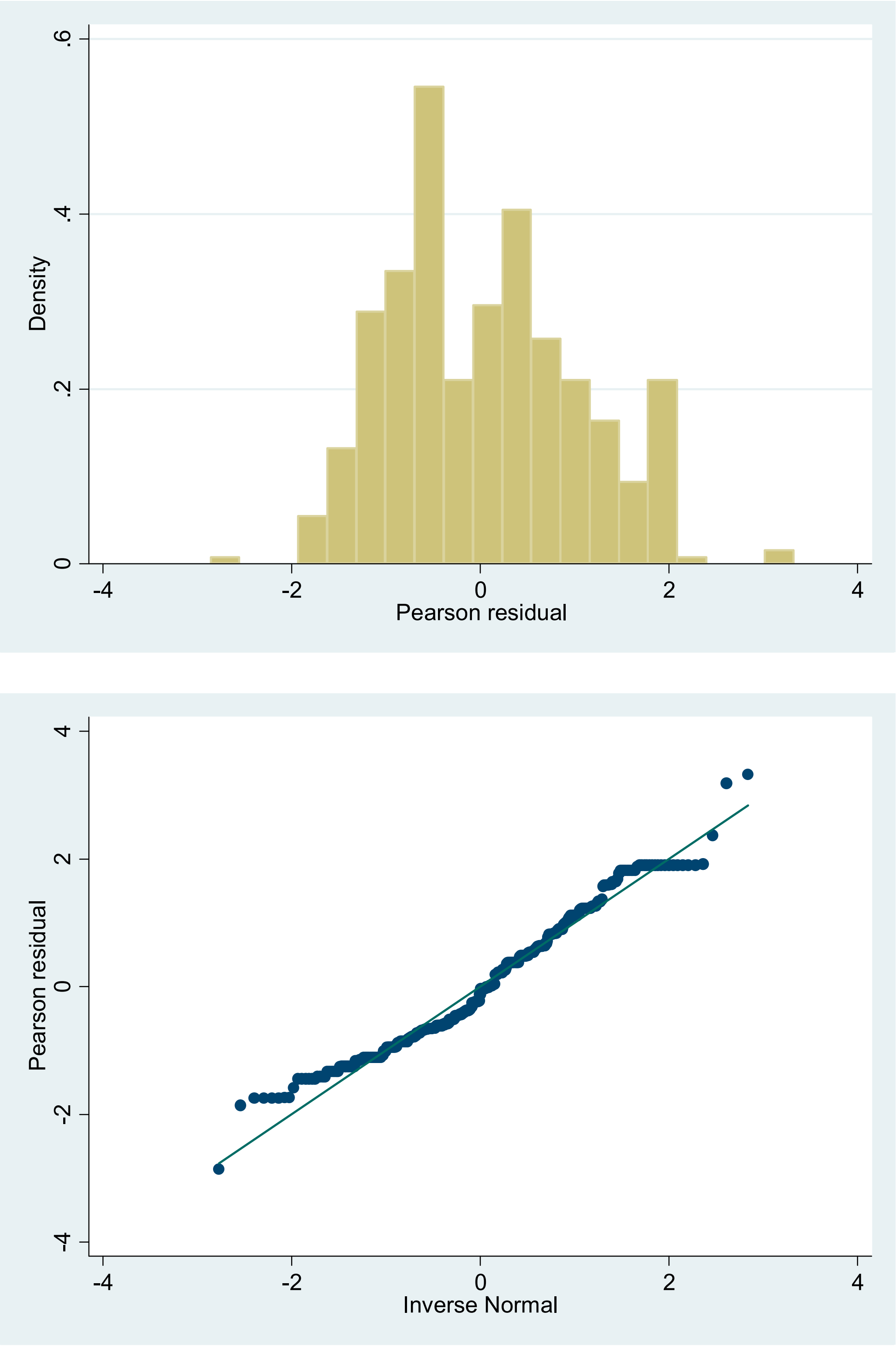

Figure S3. MBI_EE linear regression

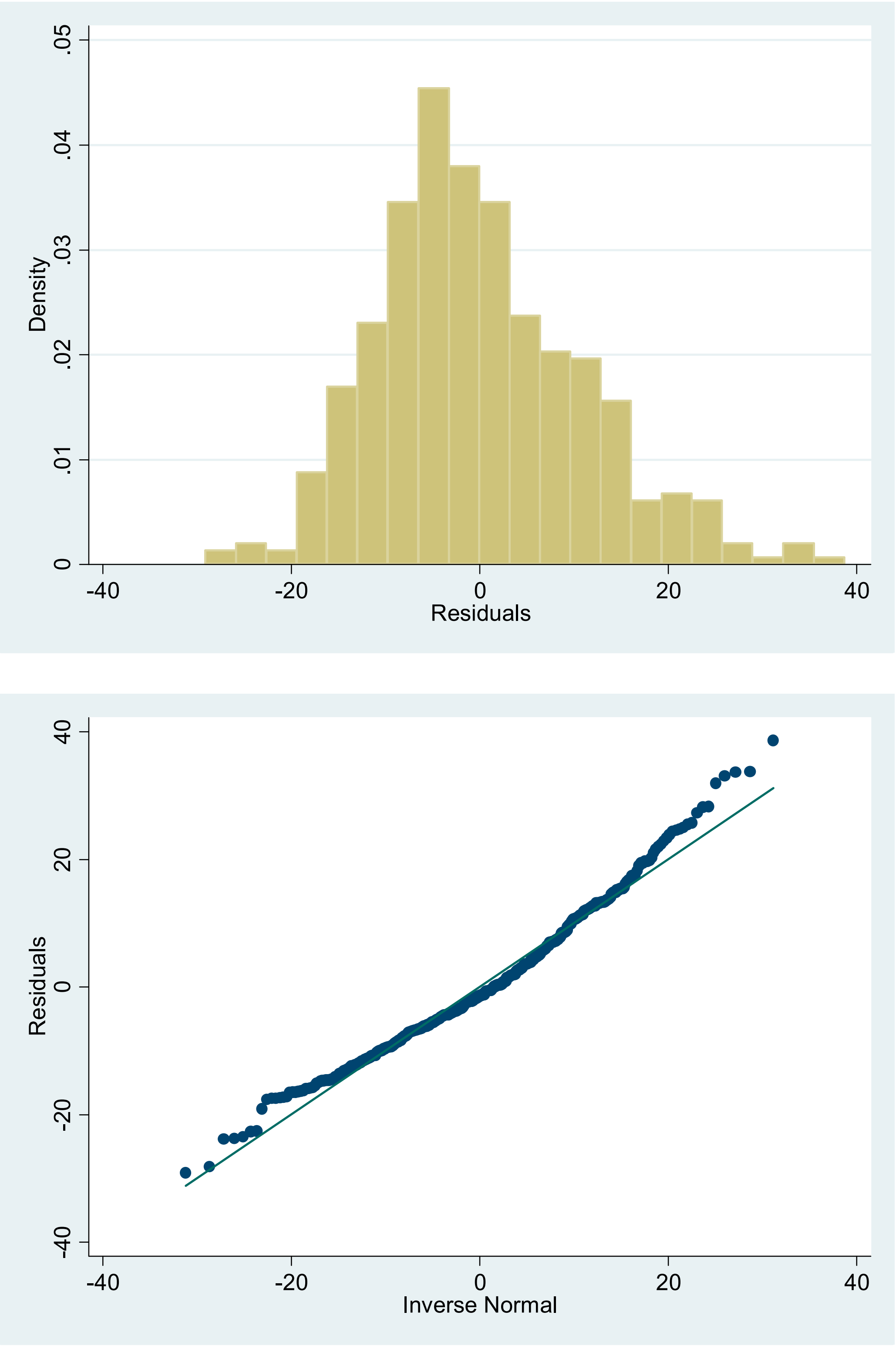

Figure S4. MBI_PA linear regression

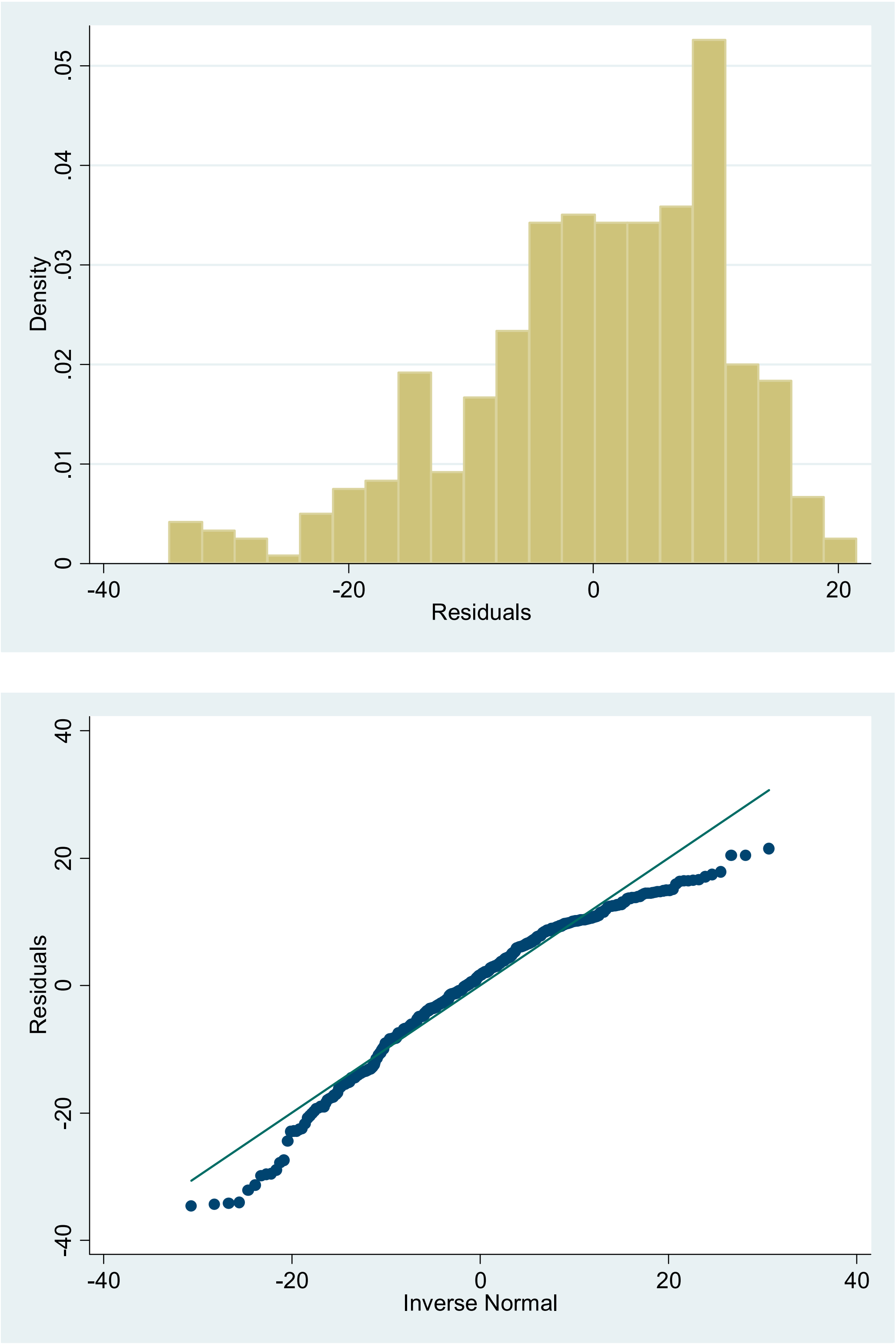

Figure S5. MBI_DE linear regression

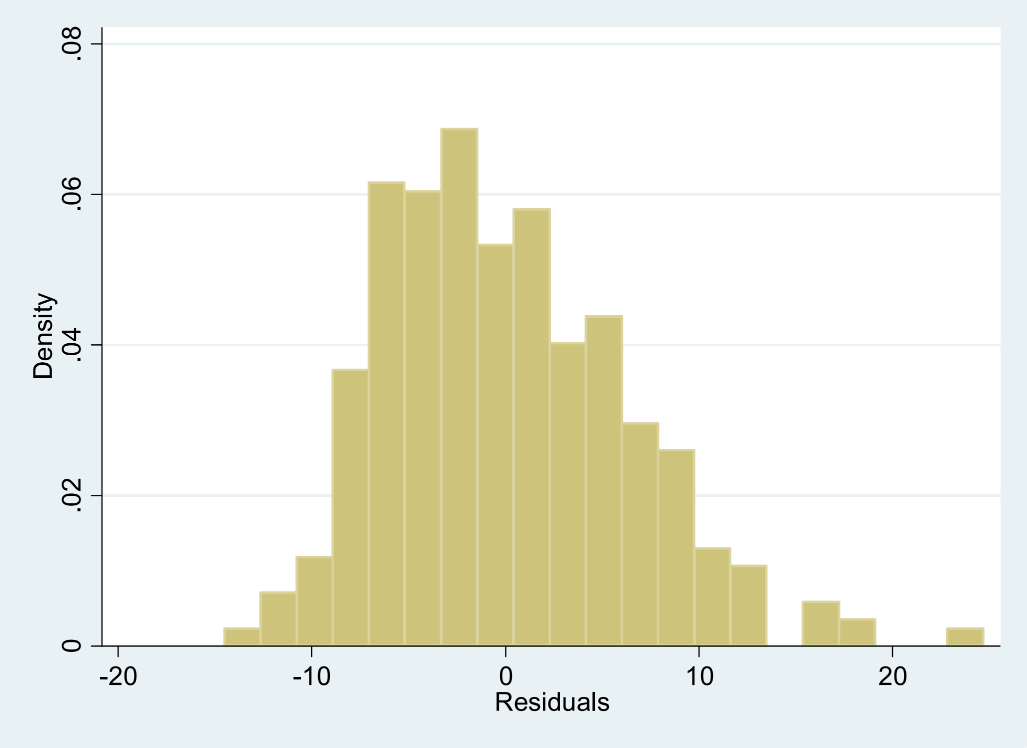

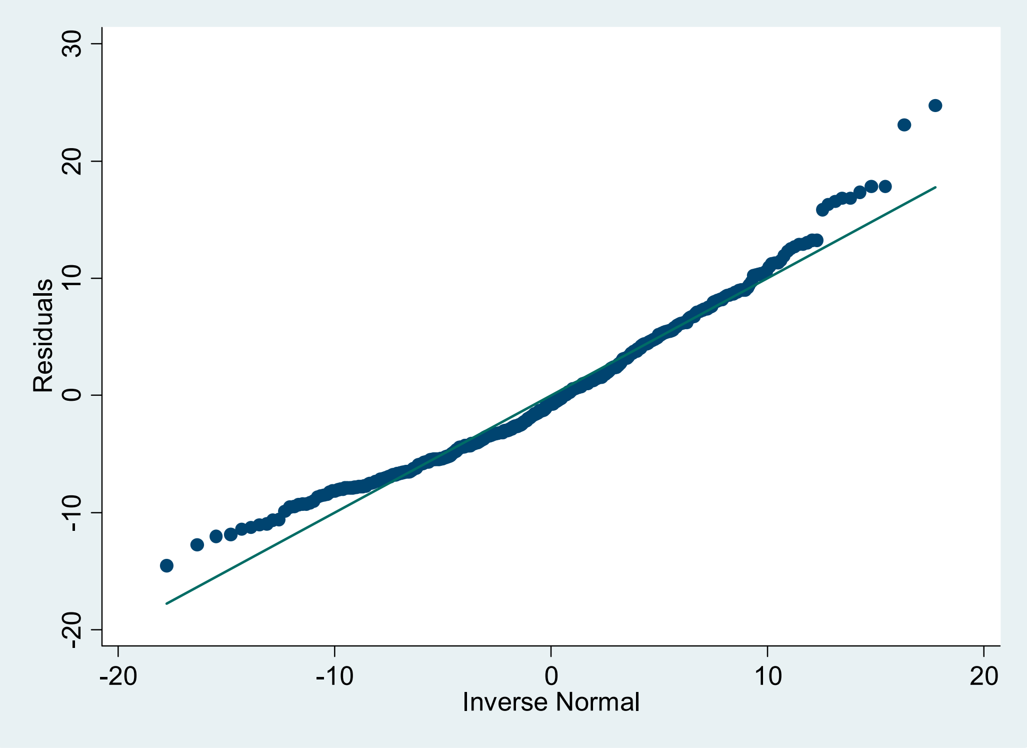

Figure S6. IESR logistic regression

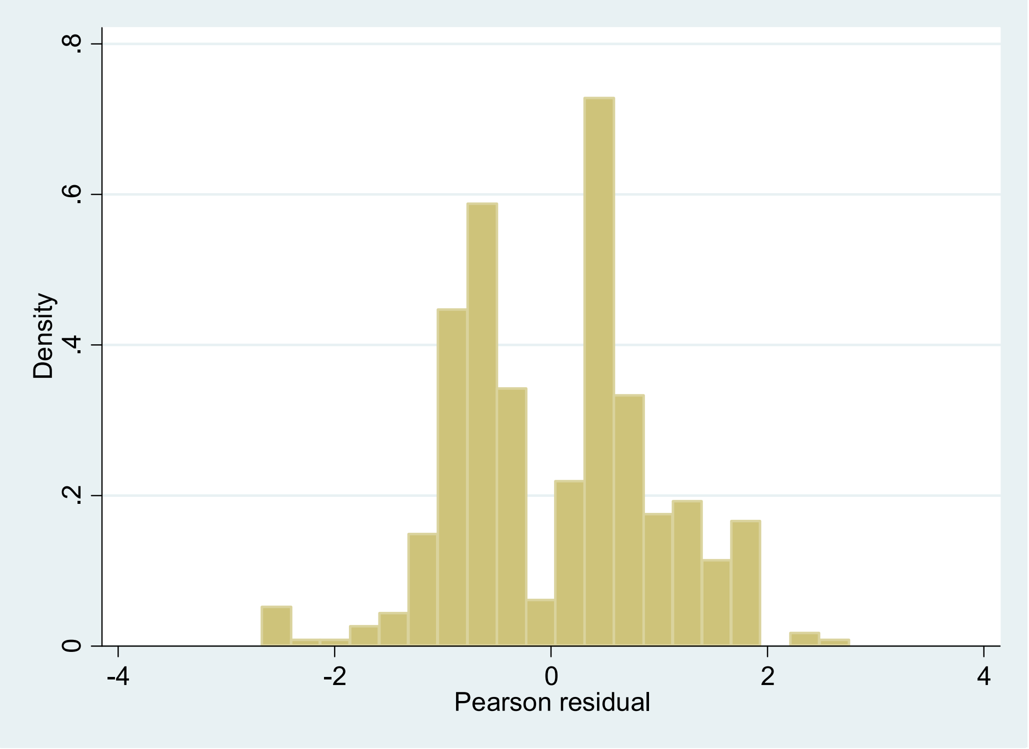

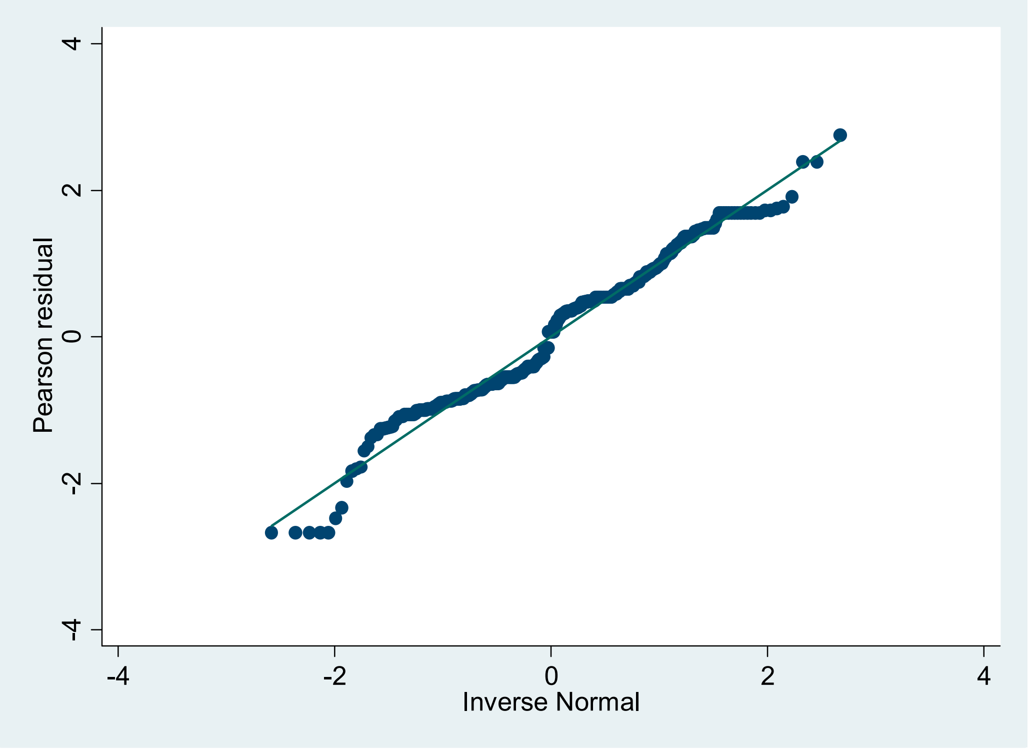

Supplement: Supplementary file 1 [file ijerph-18-02390-s001.pdf]
